# How oral traditions develop: a cautionary tale on cultural evolution from the Quechuan-speaking Andes

**DOI:** 10.1057/s41599-025-05335-4

**Published:** 2025-10-17

**Authors:** Matthias Urban

**Affiliations:** 1 Laboratoire “Dynamique du langage” (UMR 5596), National Centre for Scientific Research, Lyon, France; 2 Lumière University Lyon 2, Lyon, France

**Keywords:** Cultural and media studies, Anthropology

## Abstract

While large-scale comparative and historical analysis of folktales has largely disappeared from anthropological inquiry after the wane of diffusionism in the early 20th century, such approaches are experiencing a revival in the framework of cultural evolution. In that context, questions asked include to what extent narrative traditions are transmitted horizontally from generation to generation; influenced by practices of neighbors; and form larger packages with other expressions of culture, prominently language. Here, I explore to what extent 41 versions of a widespread story told by Indigenous Andean storytellers in the Quechuan languages show signs of having developed according to evolutionary phylogenetic mechanisms, bringing data from the underrepresented New World into the purview of the literature. The story of Juan Oso (“John the Bear”), which tells of the origins and adventures of a half-bear, half-human boy, has European roots, meaning that variation in the Central Andes only had several centuries to develop. Analyses show that the story varies in ways that can neither be explained fully by where it is told (and hence by possible “diffusion” of characteristics from region to region), nor by the Quechuan variety in which it is told (“co-evolution of language and culture”), nor, most importantly, by historical mechanisms of an evolutionary nature according to which the story might change. With reference to the ethnographic literature, I suggest that these results can be explained by the ways in which Andean storytellers recombine narrative material from stories to imbue them with new meaning that comments on local and regional social and political circumstances, and that a “rhizotic” model of development, in addition to or instead of the phylogenetic ones tested by cultural evolutionists, might be more adequate to understand how the individual versions of this story came to be told the way they are.

## Introduction

The early history of cultural anthropology is characterized by the succession of evolutionism in the 19^th^ century and diffusionism in the 20^th^. The former, now regarded as deeply problematic, would posit quasi-evolutionary stages in the development of human societies towards ever greater complexity and sophistication. The underlying assumptions of diffusionism are considered no less problematic, not least for portraying as passive recipients those people who are claimed to be at the receiving end of cultural diffusion.

Present anthropological practice has largely abandoned such diachronically oriented approaches and often also the endeavor of large-scale cross-cultural comparison, instead preferring more particularistic approaches that emphasize the active role of individuals in shaping and reshaping cultural traditions. When it comes to oral traditions (the topic of this contribution), attempts at comparative historical interpretation, especially when parallels in content form the basis for metanarratives of deep migration or contact histories that trace back thousands of years in prehistory, as posited e.g. by the “Kulturkreise” school of diffusionism, are viewed with particular skepticism.

Independently from this, however, in the field of cultural evolution, large-scale cross-cultural comparison is experiencing a revival. This is part of a broader surge of evolutionary phylogenetic models from biology into disciplines studying cultural behavior (cf. e.g., Currie, 2013 and Mesoudi, 2015 for review on this movement), including historical linguistics (e.g., Bowern, 2018), archaeology (e.g., Mendoza Straffon, 2016), the history of religion (e.g., Norenzayan et al., 2016), or the development of political complexity (e.g., Currie et al., 2010). Researchers working in the framework of cultural evolution have begun to apply methods originally developed to model biological evolution to oral traditions in an effort to trace the origins and development of folktales. Based on correlations with language phylogenies, cultural evolutionists have claimed to trace the origins of some folktales up to the Bronze age (Graça da Silva, Tehrani (2016)). Some studies also employ so-called ancestral state reconstruction to establish an “original” version of a narrative from which attested version are derived. In other words, cultural evolutionists are replicating core concerns of earlier comparative traditions in anthropology from the 20th century, and their studies resonate in particular with the historicist-diffusionist “historical-geographical” school of folktale analysis (to be reviewed in section 2).

In this contribution, I study versions of a well-known folktale from the Quechuan-speaking Andes in South America that tells of the history and adventures of a character named Juan Oso (or “John the Bear”). While having European origins, in the Americas the story has acquired distinct new elements that reference Indigenous mythology and belief systems and reflect life of Indigenous people under European rule. Anthropologists have recorded local variants of this tale at dozens of places in the Central Andes in which different Quechuan varieties are spoken.

Taking up a desideratum expressed in the literature on Andean folklore in connection with the Juan Oso story (Weber, 1987), I aim to shed light on variation in how the tale is told. In spite of a wide range of sources being available, such a comparative treatment is so far lacking.

I study aspects of the story from both qualitative and quantitative perspectives. Doing so, I tie this case to a (hopefully) more generally relevant exploration of the question to what extent variation in how the story is told reflects processes and patterns commonly invoked in quantitatively oriented literature in the cultural evolution framework: these include the question whether certain motifs are typical for certain regions of the Central Andes (spatial structure which would usually be attributed to “diffusion” in the relevant literature); whether the versions of the story recorded in different localities are related to the different Quechuan varieties in which they are told (under a scenario of “co-evolution” of language and culture); and whether they reflect transmission under conditions that are characteristic of evolutionary systems (and hence showcase evidence for evolutionary mechanisms in culture itself). I employ methods of data analysis that are similar to those used in relevant studies on the development of folktales in a cultural evolution framework, including measures of spatial autocorrelation, correlations with linguistic distance, and metrics of phylogenetic signal, and apply these to a coded dataset that reflects regional variation in the Juan Oso stories as recorded at different places of the Central Andes. The Juan Oso story as told in the Central Andes has several characteristics that make it well-suited as a case study: first, while having European origins, the story has acquired distinctively Andean characteristics, and thus contributes to remedying an emphasis on Eurasia, or even Europe more narrowly, in the available sources for large-scale studies. Second, claims for remote origins of folktales made in the literature are typically based on mapping stories onto dated language phylogenies. The Juan Oso story can open up more direct perspectives on the question what magnitude of variation we may expect in folkloric material after a certain amount of time has passed: assuming that a uniform version of the story was brought to the Americas from the Iberian peninsula, all variation observed in the Central Andean versions has had a maximal time horizon of approximately 500 years to develop.

In section two, I provide more background on the application of quantitative and phylogenetic analysis to cultural data, especially to folktales and mythological material. In section three, I give more information on the Juan Oso story and its characteristics, paying particular attention to variation in the plot of the story across the Central Andes. I then analyze the dataset, which, as I discuss in section four, I have prepared in a way that is parallel to the way tales are broken up into constituent motifs in the so-called “historical-geographical method” of folktale analysis and the cultural evolution approaches that continue its tradition. In section five, I show that, while there are some regionally salient characteristics in the way the tale is told in the Central Andes, overall there is no strong geographical variation to how the tale was recorded (no evidence for “diffusion”); there is no appreciable evidence that the structure of the tale co-varies with the structure of the Quechuan varieties in which it has been told (no evidence for “language-culture coevolution”); and with possible local exceptions, overall, there is no clear phylogenetic signal in the dataset that would indicate that the story was passed down from storyteller to storyteller through time and evolved as certain plot elements were transmitted in that process while others were lost (no evidence for “cultural evolution” in a broad sense).

In section six, I then articulate the results with ethnographic perspectives on Andean traditions of storytelling that, like elsewhere, rely on the creative agency of storytellers to imbue traditional material with new meaning in and for the particular local, regional and national contexts in which stories are told, but in particularly Andean ways. I suggest that these traditions are relevant in several regards that relate to the methodological and conceptual assumptions of quantitative analysis of folktalkes as practiced in the cultural evolution framework: first, if recorded versions of the story to a significant extent reflect storyteller’s ad hoc engagement with narratological material in a creative practice in which motifs are altered, inversed and recombined, individual recorded versions give the potentially misleading impression that they reflect “the story” as told in a particular locality whereas they may be better viewed as one product of a larger creative practice among other, equally valid ones. Second, that storytellers engage with motifs of tales, and in some cases even recombine entire tales to provide reflections on events in the community as well as regional social, political, and economic conditions under which people live, provides a perspective on the development of stories and their adaptiveness that is not commonly reckoned with in studies of narrative traditions in the cultural evolution framework. Third, evolutionary phylogenetic reconstruction requires the specification of underlying models for how stories, and variation between them, are generated; however, to my knowledge, these models do not capture these particular ways stories are crafted in places like the Central Andes.

## Background on research traditions

The study of folktales and mythology has a rich and chequered history that I cannot hope to adequately summarize in any detail. Some of the oldest traditions of research, dating to the 19th century, had a strong comparative, historicist outlook and tried to explain similarities in folktalkes and myth in a now widely discredited diffusionist framework.

One particularly influential tradition that emerged from these beginnings is the so-called “historical-geographical” school, which developed in Scandinavia in the late 19th and 20th century in the context of the detailed study of the folklore of Finnic people (Krohn, 1926). The “historical-geographical” school recognized spatially structured variation in folktales and myths, and used this variation to work its way backwards to infer an area where a folktale or myth had originated and to reconstruct its original form, often with the idea that change leads to degeneration of “pure” original versions (Dundes, 1969). This tradition also established the beginnings of a more large-scale system to classify folktales (mostly of Europe and Eurasia) into various “types” based on their plots (Aarne,^ 1908^); a revised and expanded version is known as the Aarne–Thompson–Uther Index (Uther, 2004).

Some leading anthropologists of the time were sympathetic with the enterprise (Franz Boas, 1912: 258, for instance, remarked that it might be possible, through analysis of the various American versions of the tale of Juan Oso that I analyze here, to trace them back to their ancestral European forms and to, in his words, “clear up lines of importation”). However, this essentializing and at the same time atomizing approach has been heavily criticized (Jacobs, 1966), and by now largely abandoned as scholars have come to emphasize the fluidity of folktales; the cultural and political contexts that condition them; and the creative power of storytellers as opposed to broad spatial patterns in variation that, as “historical-geographical” scholars would argue, reflect descent with modification from a common ancestral archetype.

At the same time, however, the historically oriented and comparative outlook of the “historical-geographical” school chimes with contemporary approaches to culture in the framework of cultural evolution (e.g., Boyd and Richerson, 1985; Boyd et al., 1997; Mesoudi et al., 2004). Generally, these approaches attempt to model tangible and intangible cultural products –which may be, among other things, aspects of artifacts, languages, or in this case traditional narratives– as the result of evolutionary processes that, like biological evolution, are characterized by variation (there is more than one way to make an artifact, more than one way to express oneself, and more than one way to tell a tale), involve transmission of information from generation to generation (inheritance), possibly under conditions of selection (as when, e.g., a particular way of making an artifact is more efficient or leads to better quality and hence ousts other techniques over time). To study aspects of culture in this way, cultural evolutionists typically parametrize and thereby atomize their objects of study. In the case of wind instruments across cultures, one might investigate the length of the tubes and their number; the number of holes in each, etc. (cf. Aguirre-Fernández et al., 2021). In the case of folktales or myths, the units of analysis would be the presence or absence of particular plot elements, protagonists and their traits, etc. In each case, the result is a matrix of characters that is the analogue to a matrix of characters that indicate biological variation, e.g., the presence or absence of a mutation at a particular locus of the genome.

Cultural evolutionists working on folktales have investigated whether versions of folktales are particularly similar among peoples who live in proximity to one another (which is typically attributed to “horizontal transmission” or “diffusion” of folktale traits between neighboring people, similarly to 19th century diffusionist theory). Some studies furthermore investigated whether folktales are transmitted as part of a more general “cultural package,” where typically linguistic affinity is used as a proxy for “culture” as a whole. More generally, they are interested in the question to what extent observable variation between different versions carries a phylogenetic signal that reflects transmission under conditions of variation (and possibly selection). Some studies also employ so-called ancestral state reconstruction to establish an “original” version of the narrative from which attested versions are then derivable through evolutionary processes (and hence, operationalize the traditional goal of the followers of the “historical-geographical” method using powerful contemporary algorithms). In sum, cultural evolutionists study questions that are strikingly similar to those that were central to the scholars of the “historical-geographical” school in the early 20th century on the basis of an intellectual framework inspired by, and with methods derived from, biological evolution.

Results are mixed. European and Arctic folktale data have not been found to show strong evidence for developing according to evolutionary processes, suggesting that geographical proximity (and presumably, interaction between people living in neighboring areas) is a factor that has more influence on folktales than their “vertical” transmission from generation to generation here (Ross et al., 2013; Ross and Atkinson, 2016). Correlation with language disappears when controlling for geography in one study (Ross and Atkinson, 2016), suggesting that narrative traditions and the languages in which they are told develop within different time frames, or that the link is obscured by unrelated processes (such as, indeed, possible “diffusion” of narrative material from speech community to speech community).

On the other hand of the spectrum, Tehrani (2013) suggests that the fairytale Little Red Riding Hood shows evidence of having developed in a manner that is consistent with the assumption of cultural evolutionary processes. Graça da Silva and Tehrani (2016), moreover, relate folktale variation to independently established language phylogenies and find a strong relationship between the two which is not confounded by geographical proximity, leading them to posit a language-culture-coevolution scenario that shaped both the content and motifs of a set of folktales as well as the languages in which they are told. What is more, since in this case the languages are Indo-European, a widely diversified language family that has roots deep in the Bronze Age, they make the astonishing claim that particular tales, like ‘The Smith and the Devil’, likewise, have their origin in the Bronze Age, commensurate with the linguistic phylogeny.

## The tale of Juan Oso in the Andes

In this study, I will be concerned with variants of a widely known story about a character known as Juan Oso (or “John the Bear”).

This story of the adventures of a half-bear, half-human being has origins in the Old World, and is told across much of Europe and beyond. The European versions are usefully summarized in Panzer (1910). Various versions from the Iberian peninsula have been recorded in the 15^th^ or 16^th^ century; it is from versions like these that Indigenous American versions must be derived. Boas (1912: 254), for instance, discusses Mexican Indigenous version. A first Andean version was recorded by the Spanish chronicler Cabello Valboa in Ecuador before 1586, showing that the story was known in the Andes already in the first decades after European conquest in the early 16^th^ century. While having European origins, this story is widely known among Indigenous people of the Central Andes, and has been recorded in Quechuan languages and other Indigenous tongues at various localities.

The Andean versions of the story tell of a girl or a young woman who encounters a bear. The background of the woman and the setting of the encounter vary. In some versions, she is a shepherdess, in another, a farmer’s girl, in yet another, she is the daughter of an eremite mother who, after the death of her husband, lives in the forest. In one version, she is collecting firewood when encountering the bear, in another, she is bathing, in yet another, she is lost in the mountains, etc. The woman is then abducted by the bear and subsequently involuntarily lives together with him in his lair (in one version though, she goes with him voluntarily). Most stories tell of one or more attempts to contact relatives or other humans that represent civilized life in human society. These failing, the woman eventually gives birth to one or more children, among who the protagonist of the story, Juan Oso. After varying preludes she and her child(ren) ultimately manage to escape from the cave and reconnect with the sphere of civilization, while the bear is subsequently killed in different ways.

There are a few versions of the story in which at that point Juan Oso is killed as well; however, in most versions, at this point a clearly distinguishable “second act”, which narrates the further fate of the boy and his troubles integrating into human society, commences. In many versions, the half-bear, half-human boy is given into the custody of a priest to help improve the antisocial or violent behavior that results from his bear heritage, in particular at school. This priest, however, is not always well-meaning, and sends Juan Oso on fool’s errands to carry out various dangerous tasks, secretly hoping that he would not survive them. In some versions, this is successful. In many others, however, the bravery and strength of Juan Oso help him survive the various ordeals, as when he captures some ferocious bulls who the priest speculates would kill him, and instead kills the bulls thanks to his bearish strength (or else brings them to the priest). Mastering the ordeals in some versions has a cathartic effect on Juan Oso, and he ultimately succeeds in integrating into human society.

The basic constellation Juan Oso finds himself in is that of an in-between, who neither belongs to the sphere of untamed nature nor to the sphere of human society. The boy’s difficulties adapting to life among humans are already present in Iberian versions (Boas, 1912: 254), so that in general these cannot be attributed to Indigenous transformation. At the same time, however, bears as mediators between different stages and roles in social life are already known from the pre-Columbian Andes (Paisley and Saunders, 2010) and thus pre-dispose the Juan Oso story to be adapted to an Indigenous Andean context. Indeed the story has been argued to relate to the “colonial experience” (Allen, 2002: 72) of Indigenous people of the Central Andes, who found themselves thrown into a European-dominated society which systematically disadvantages and marginalizes them, but to which they are nevertheless forced to adapt. Also more minute aspects of the story as told in the Central Andes lend it a characteristic coloring. One salient change vis-a-vis European versions (among others) is that an episode in which Juan Oso fights with a demon to liberate royal virgins from the underworld has been replaced with a confrontation with a *condenado*, a typically Andean undead creature that independently is the subject of many a tale as well (Cippoletti, 1983).

The versions I will be dealing with have been told in languages of the Quechuan family by Indigenous storytellers. Quechuan is one of the largest Indigenous language families of the Americas both in terms of its geographical extent, which ranges from southernmost Colombia into Northern Argentina, and in terms of the number of speakers. While use recedes in favor of Spanish almost everywhere throughout its range, there are still several million speakers when all varieties are taken together. Quechuan is a language family, not a single language. While in many parts of its extent the family has the characteristics of a dialect continuum, local variation in vocabulary, pronunciation and grammar is large enough that speakers of highly divergent varieties cannot communicate with one another. Internal variation has been compared impressionistically to that of the Romance languages, perhaps minus the divergent French; the time passed since the onset of the divergence of ancestral Quechua to become a language family is thought to be approximately 1500 years (Heggarty, 2007). While major dialectal configurations thus were already in place at the point of European contact, the 500 years of historical development since then are a significant part of the total time period in which Quechuan languages have been diversifying, and many of the characteristics of present-day varieties, such as coda lenition in Southern Quechua, were still in the process of being configured when Quechuan speech is first attested (Mannheim, 1991). This gives us an unusually close match between the time frame for diversification processes in European oral traditions and the languages in which stories are told. This affords opportunities to investigate to what extent (if any) folktales and other narratological material is transmitted together with larger cultural packages for which language has usually been taken as the most readily available proxy.

## Data sources and representation

Data come from two main sources: The first is Morote Best’s (1988[1957]) classic collection, which contains 26 regional versions of the Juan Oso story from all over the Quechuan-speaking Peruvian Central Andes. A focus is on the department of Cuzco in southern Peru, which alone is represented by 15 distinct versions. Importantly, some of the versions were collected at one and the same place, providing an important opportunity to assess intra-site variation (Morote Best also included two stories, from Carhuas in northern Peru and Omacha in Southern Peru respectively, which he considered related to the tale of Juan Oso but still sufficiently different to not treat them on par with the others. This is symptomatic of a key criticism of the “historical-geographic” school and the folktale indices that have emerged from them, according to which variation is so fluid that any reified types are not warranted and arbitrary. Here, I respect Morote Best’s assessment and do not include the tales he considered deviant in my formal analysis. However, I return on this point in the discussion). Unfortunately, the stories in Morote Best (1988[1957]) are only available in Spanish translation, and are also already summarized as a metalinguistic commentary on the actual stories as told to Morote Best. The second source is the bilingual collection by Weber (1987), which has 16 additional stories, fifteen of which told in local Quechuan varieties throughout Peru, including two varieties of the tropical lowlands to the immediate east of the Central Andes (a further story was told in the Arawakan Matsigenka language). I have also included the 16^th^ century version reported by Cabello Valboa (2011[1586]); note, however, that Cabello Valboa does not frame the content as a story, but as a factual report of something he heard had happened near the village of Numbacola in southern Ecuador.

I have then isolated characteristic plot elements of the individual versions in a way that is close to what a cultural evolutionist would do. I have been guided in this partially by how Morote Best himself proceeded. For instance, Morote Best has placed emphasis on stating, for each version of the story, the background of the young woman who is abducted by the bear and the setting in which that occurs. So have I. Overall, I have identified 126 ways in which the stories differ (the characters for later formal analysis). My coding indexes the presence or absence of individual features. Informally, I have grouped these traits into a smaller number of categories that correspond to different episodes of the story as it unfolds: the circumstances of the encounter with the bear; how the abduction to the cave proceeds; what events transpire while bear and young women live together in the cave; the young woman’s failed contact attempts with society; the birth of Juan Oso (and other offspring, in some versions); the preparations the woman makes for the flight; the particulars of the flight itself; the events immediately thereafter, in particular relating to whether, and if so, how, the father bear learns of the flight and pursues his wife and children; the circumstances of the death of the father bear (where present); and the circumstances of the death of Juan Oso for those versions of the story that end here. For those where this is not the case, further categories relate to Juan Oso’s troubles adjusting to human society; his further fate, including prominently the custody of the Catholic priest; the various ordeals he has to undergo; the setting and unfolding of the battle with the *condenado*, if present; and Juan Oso’s further fate after the main events of the story have come to a close.

A characteristic of the dataset is that many of the events are unique to a single version of the story and do not appear in any other. Another characteristic is that certain events, because of preceding events, logically cannot happen in the universe of some versions, for instance, when the protagonist has died. To distinguish this situation from characteristics or events that *could* be present or happen, but do not, I have in principle coded events that are logically impossible in the universe of a particular story as being not applicable (i.e., NA) than non-occuring (i.e., 0). This means that for some versions of the story –such as those in which Juan Oso dies before undergoing the various ordeals—a large number of characters are missing. Note also that the *condenado* episode is often one of the ordeals Juan Oso is assigned to, but is so salient that it sometimes also occurs out of that context. Therefore, I have coded all ordeals as NA if Juan Oso is not handed over to the priest and he or the mother plot to get rid of him, except for the *condenado* episode which I consider a conceptually separate motif complex.

Together, both procedures lead to a sparse fully binarized dataset with a large number of uninformative traits. While the Jaccard distance, which I will rely on for most analyses to compare versions of the story, is based on the number of plot elements versions share and hence not directly sensitive to shared absences, I also prepared a second version of the dataset, for which I removed uninformative characters and recoded some sets of fully binarized characters as single multistate characters. In this version of the dataset, for example, the background of the woman is captured by a single multistate character with the following values: [1] the woman is a shepherdess; [2] the woman is a farmer’s girl; [3] the woman is an orphan; [4] the woman is the daughter of a woman who lives alone in the forest; and [5] the woman is married and already has a child when the bear abducts her. I have induced dissimilarity scores for the multistate dataset by Hamming distances calculated using the hammingdists function of the cultevo R package (Stadler, 2018).

To look into the question whether the structure of the story co-varies with linguistic differentiation (as posited by cultural evolutionists who ponder a “cultural package” that is passed on from generation to generation in conjunction), I have created a second dataset that captures salient variables in which the varieties of Quechuan in which the stories are told differ. Quechuan is a shallow language family, and individual varieties are typically distinguished by only a relatively small number of diagnostic parameters. The dataset captures these parameters of variation (drawn from the pertinent literature: Landerman, 1991, Cerrón-Palomino 2003 [1987], Adelaar with Muysken, 2004). They concern the pronunciation and organization of the sound system (phonology) as well as the grammatical elements that speakers have at their disposal to express relationships between different parts of a sentence or phrase and their form (morphology). The parameters of variation are typically framed in diachronic terms to trace the internal diversification within the language family. For instance, in some Quechuan varieties, an initial /h/ in words is lost, so that for instance the word *hina* “like, alike” becomes *ina*, whereas in others the /h/ remains pronounced. All in all, there are 25 parameters of variation in the dataset, which were coded based on published sources (Adelaar, 1977; Parker, 1976; Coombs et al., 1976; Cusihuamán, 1976; Quesada Castillo, 1976; Soto Ruiz, 1976; Landerman, 1991, 1994; Taylor, 1994, 1996; Cerrón-Palomino, 2003[1987]; Adelaar with Muysken, 2004). A small number of missing values were imputed using the missRanger package in R (Mayer, 2024). The mapping from story to variety is not isomorphic, but often many-to-one: for instance, the stories recorded by Morote Best in the department of Cuzco are all told in a single variety that showcases only minimal subdialectal variation that cannot be captured systematically.

**Fig. 1:**
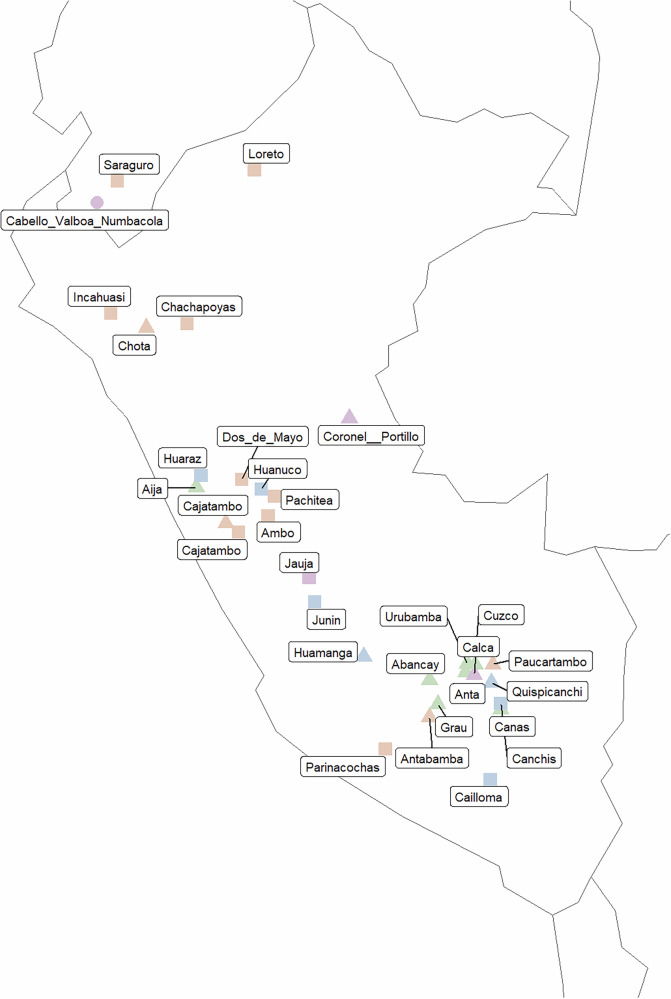
Map of Peru and Ecuador with locations at which versions of the Juan Oso story were recorded. Different shapes represent different sources (circle: Cabello Valboa [1586]^2011^; square: Morote Best [1957]^1988^; triangle: Weber 1987), different colors give an impression of the plot based on splits in a NeighborNet graph represented in Fig. 5. Map created using the sf (Pebesma 2018; Pebesma and Bivand 2023), rnaturalearth (Massicotte et al., 2023), rnaturalearthdata (South et al., 2024), ggplot2 (Wickham 2016; Wickham et al., 2024), and ggrepel (Slowikowski et al., 2024) R packages.

A third dataset contains the geographical coordinates of the places in which stories have been recorded. In Morote Best (1988[1957]), this is resolved only at the level of the province, but the precise communities in which stories were actually recorded are not mentioned. I have therefore usually used the coordinates of the capital (administrative seat) of the province. In Weber (1987) the communities in which the stories were recorded are identified more precisely. Figure 1 plots the locations as represented in this dataset; different symbols distinguish the three different sources (Cabello Valboa, Morote Best, and Weber), while colors represent the place of the respective versions in a NeighborNet analysis reported in Section 5.3 to give some visual idea as to how different individual varieties are with regard to the plot.

## Results

### Weak geographical structure

Regional variation in the stories is apparent. As Weber (1987: 14) notes, many northern versions end with the death of the father bear, whereas this part of the story is not present in San Martín in the lowlands and in Cajamarca. In contrast, the battle with the *condenado* is a trait of the tale as told in Central and Southern Peru.

**Fig. 2:**
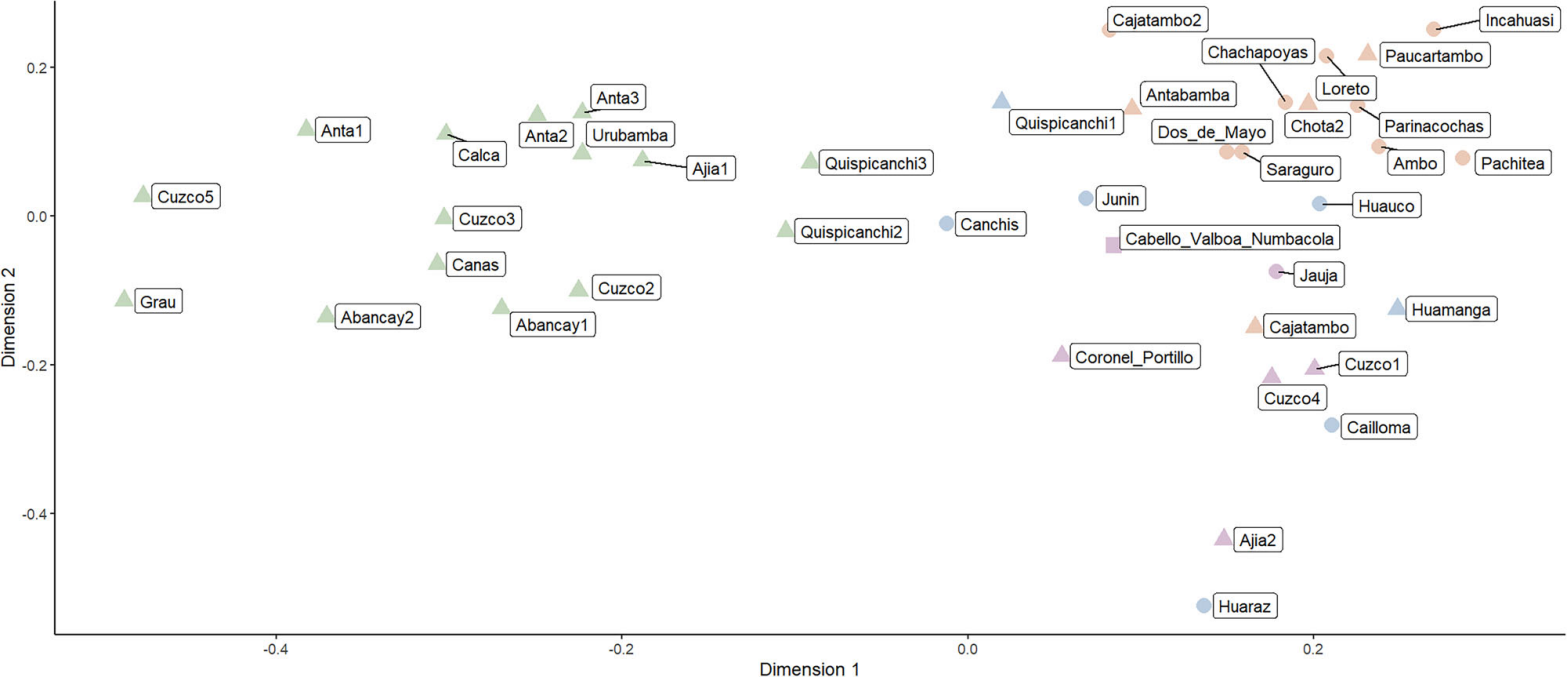
Multidimensional scaling plot of variation in the story, based on 126 distinct plot elements. Created using the ggplot2 (Wickham 2016; Wickham et al., 2024), and ggrepel (Slowikowski et al., 2024) R packages. Different shapes represent different sources (circle: Cabello Valboa [1586]^2011^; square: Morote Best [1957]^1988^; triangle: Weber 1987), different colors give an impression of the plot based on splits in a NeighborNet graph represented in Fig. 5.

Inspection of a multidimensional scaling plot that represents the structure of the tale (based on the fully binarized coding) in two-dimensional space (Fig. 2) likewise suggests some geographical patterning.

For instance, a group of versions from the Cuzco department in Southern Peru have low values on Dimension one, whereas the version from Huaraz and one of the versions told at Ajia, both in Northern Peru, have singularly low values on Dimension two. On the other hand, other versions also told in Cuzco are virtually on the other end of variability along Dimension one, and a further version recorded at Ajia is likewise very distant from the one just mentioned. In other words, stories as told in the same community or in communities that are very close to one another, can be heterogeneous enough so as to virtually exhaust the space of variation across all analyzed stories taken together.

To investigate geographical variation more formally, I have carried out a distance-based Redundancy Analysis using the rda() function of the vegan R package (Oksanen et al., 2024). For this analysis, I have treated logically impossible events as zeros rather than missing data to avoid data leakage. In this analysis, the geographical coordinates of the communities at which stories were recorded explained only 7.6% of variation across the fully binarized dataset, suggesting that there is little geographical structure. However, a permutation tests for the joint effect of constraints still returned a significant effect under permutation against a reduced model (Variance 0.6308, *F* = 1.5734, *p* = 0.01). When employing multistate coding, in contrast, explained variation increases to 23%, but conventional significance thresholds are not reached (Variance 1.6175, *F* = 1.3452, *p* = 0.19).

**Fig. 3:**
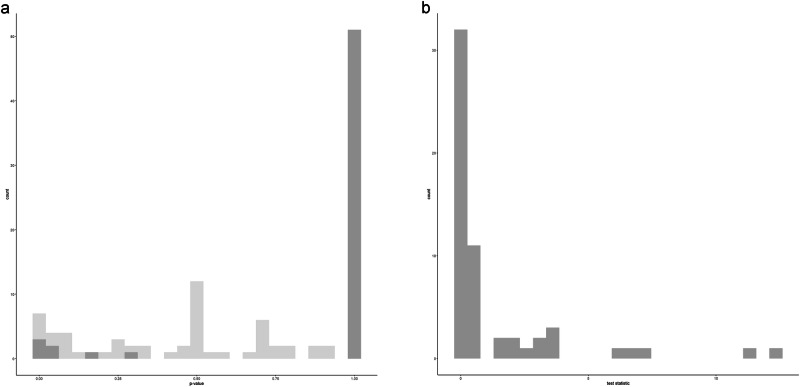
Join-Count analysis. **a** Test statistic for individual plot elements. **b** Associated p-values before and after correction. Created using the ggplot2 (Wickham, 2016, Wickham et al., 2024) R package.

To investigate spatial variation in the data further in light of these inconsistent results, I looked at variation in the individual plot elements and their occurence in space using Join-Count analysis as implemented in the spdep R package (Pebesma and Bivand, 2023; Bivand et al., 2024). Figure 3 plots the resulting test statistics for each fully binarized plot element (right) as well as associated uncorrected *p* values (right, light gray) and *p*-values after Bonferroni correction as implemented in Base R’s stats package (right, dark gray).

**Fig. 4:**
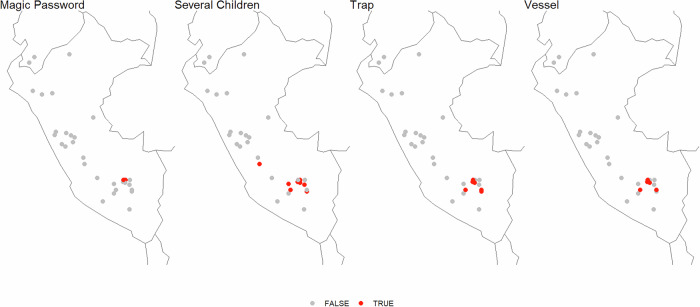
Panel plot with maps displaying versions of the story (red dots) which feature significantly clustered story elements according to Join-Count statistics after correction for multiple comparison. Map created using the sf (Pebesma, 2018; Pebesma and Bivand, 2023), rnaturalearth (Massicotte et al., 2023), rnaturalearthdata (South et al., 2024), and ggplot2 (Wickham, 2016; Wickham et al., 2024) R packages.

The low values of the test statistic and the near-normal distribution of uncorrected *p*-values suggest that the distribution of the majority of features is not clustered in geographical space, consistent with the low explanatory power of geography in distance-based Redundancy Analysis. However, there are four plot elements which are significantly clustered geographically after Bonferroni correction. In the relevant stories, there is a magic password that opens and closes the bear’s cave; the woman gives birth to Juan Oso and one or more further children; a trap is prepared to catch and kill the father bear after the woman’s flight; and in the relevant versions, that trap specifically consists of a vessel with boiling water (with some disguising covering on which the bear is invited to take seat). The latter three features are those with the highest feature-scores in distance-based Redundancy Analysis, suggesting that both types of analysis pick up on the same signal which pertains to a small number of plot elements. As Fig. 4 shows, the stories with these characteristics are strongly clustered in the Cuzco department (though, importantly, in the same area stories in which none of these plot elements appear have been recorded as well).

In most versions of the story, the trap placed to catch and kill the father bear is in fact a cauldron or another vessel, so that these two elements are not independent from one another (only in a small minority of stories a different trap is prepared). On the other hand, there is no logical connection between the women having several half-bear, half-human children and the fact that the bear is trapped and killed at a later point of time. Yet, it is the same stories that show these characteristics jointly, making them candidates for potentially clade-defining shared innovations.

The magic password, on the other hand, conspiciously evokes Ali Baba and the Forty Thieves, where “Open Sesame!” is the magic phrase that opens a cave in which thieves are hiding, suggesting strongly that this element has been incorporated by Andean Storytellers familiar with that tale.

Both observations give a first idea of how it is that folktales in the Central Andes can develop. I will come back to both later.

Results for the dataset employing multistate coding are comparable. Three features remain significant at *p* < 0.05 after Bonferroni correction: the use of a magic password to open the cave; the circumstances of the father bear’s death (which relates to the abovementioned placement of traps); and complaints about Juan Oso’s behavior made by villagers or his classmates’ parents.

In sum, rather than geographically structured gradient variation that would be expected under “isolation by distance”-like mechanisms of diversification, what objectively identifiable geographical structure there is to variation in the Juan Oso tale in the Central Andes is mostly created by commonalities in some versions of the story as told around Cuzco, which is densely represented in particular in the Morote Best collection. Counterbalancing this, on the other hand, is the fact that sometimes versions recorded at the same place differ to such an extent that the variation between them comes close to the maximum observed in the entire dataset. This reinforces the impression that factors other than geographical proximity play an important role.

### No evidence for language-culture coevolution

To investigate to what extent variation in the Juan Oso story is related to differences between the Quechuan varieties in which it is told, I next quantified the association between linguistic distance, geographic distance, and variation in the Juan Oso story by calculating correlations between distance matrices using Redundancy Analysis and Partial Redundancy Analysis.

Family-internal diversity in Quechuan is not predicated on geographical distance when the family is contemplated in its entirety (Variance = 2.7795, *F* = 0.985, *p* = 0.53) (for this analysis, the Glottolog 5.0 classification (Hammarström et al., 2024) was used, and coordinates were taken from this source; where more fine-grained variation not captured by the Glottolog classification is reflected in the dataset I have used coordinates from the Juan Oso story dataset). This is expected, since the Northern and Southern Quechua varieties are generally thought to be more closely related to one another than either is to the Central Quechua varieties.

Furthermore, linguistic differentiation within the Quechuan family is also unrelated to geographical variation in the Juan Oso story. This is true when linguistic differentiation is assessed to predict variation in the story directly (Redundancy Analysis: Variance 2.9776, *F* = 1.0493, *p* = 0.33, with multistate coding: Variance 9.3685; *F* = 1.1526, *p* = 0.20) as well as when this is done while accounting for geographical distance (Partial Redundancy Analysis: Variance: 2.7795, *F* = 0.985, *p* =0.53; multistate coding: Variance 8.8134; *F* = 1.0767; *p* = 0.34)

On the one hand, this result is not surprising when considering that the Quechuan family began to diversify well before the European invasion, and major divisions were already in place by that time. On the other hand, several of the features that distinguish present-day varieties, such as the lenition of syllable codas or the sound change */ʃ/ > /s/, were still not in place and the current dialectal landscape was still in an active process of being configured. The relevant changes concern in particular the Southern Quechua varieties of the Ayacucho and Cuzco departments in Southern Peru and those of Bolivia, i.e., those that show some evidence for shared plot elements in the Juan Oso story that might be describable as shared innovations. The absence of the correlation suggests that either the portion of linguistic variation already in place in the 16^th^ century outweighs that part that was still being configured leading to an expectable absence of global correlation between linguistic and narrative variation; that the development of the story in Cuzco occurred in ways that were not in sync with the linguistic development; or both.

### No evidence for phylogenetic evolution

In this section I explore to what extent the data show evidence for having evolved through evolutionary processes of transmission with modification. The principal goal is thus not to reconstruct a phylogeny from the data, but rather to consider how *appropriate* it would be to reconstruct such a phylogeny.

Therefore, I have first created a NeighborNet graph (Bryant and Moulton, 2004, Bryant and Huson, 2023) using the SplitsTree App (Huson and Bryant, 2024). NeighborNets are Split Networks based on agglomerative Neighbor-Joining. They represent an exploratory phylogenetic technique that does not impose a tree structure on the data; instead, they allow to visualize conflicting reticulate signals in a network-like structure and thus in ways that a phylogenetic tree does not.

**Fig. 5:**
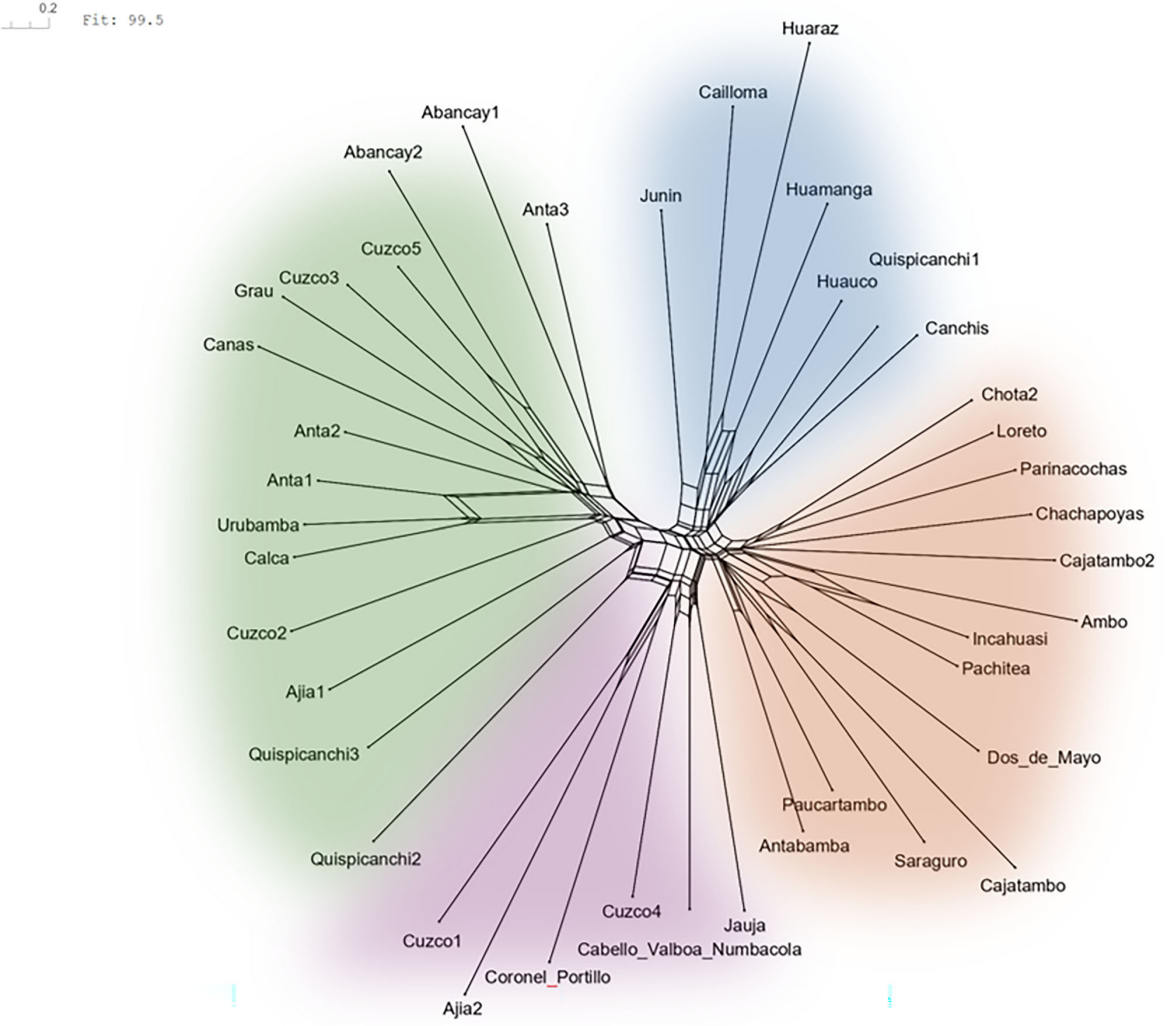
NeighborNet graph of versions of the story built upon variation in plot elements. Graph created using the SplitsTree App (Huson and Bryant, 2024); color added manually.

The resulting graph (Fig. 5) suggests that recorded versions of the Juan Oso story split into four visually distinguishable groups (manually color-coded in Fig. 5). However, each shows incompatible splits (indicated by the box-like structures at the base) that reflect signals that conflict with a phylogenetic model based on descent with modification. In addition, these four groups are not strongly distinguished from one another, but form part of a general star-shaped network with relatively little evidence for joined development of groups along phylogenetic lines. This suggests that other processes have played a significant role in the development of the recorded version of the Juan Oso story, and that a phylogenetic tree is not an appropriate representation of their relationship.

To quantify this impression, I have computed δ plots [Holland et al., 2002] using the delta.plot function of the R package ape (Paradis et al., 2004, 2024). Based on the so-called four-point condition on the distances between leaves of a tree that holds if a tree is phylogenetically completely resolved, δ is a well-known metric that allows the identification of nontreelike data arising from non-evolutionary processes. δ scores approach 0 when data are perfectly resolved in an inferred phylogeny and verge further towards 1 the less this is the case. In this case, the δ scores associated with the different variants of the Juan Oso story vary from a minimum of 0.33 to a maximum of 0.43 (multistate coding: [0.36 0.46]). δ scores are not inherently comparable across different datasets. However, a delta score of 0.26 has been interpreted as providing only “moderate” evidence for treelikeness in the context of cultural evolution of ideas on evolution itself [Fisler et al., 2020], and comparable delta scores as a signal of non-tree-like evolution in case of linguistic diversification [Yang et al., 2024].

Together, these analyses show that the processes of diversification that led to the way Andean storytellers have crafted extant versions of the story of Juan Oso are not well-describable in terms of tree-like phylogenetic evolution. These results are different from those reporting strong evidence for a phylogenetic model of the development of folktales (Tehrani, 2013). Likewise, Neighbor Net analysis shows considerably more reticulate structure, and is more similar to that obtained by Ross and Atkinson (2016) for folktale data from the Arctic.

The study of the Juan Oso story, which counterbalances the biases toward Eurasia in applications of cultural evolution frameworks to narrative traditions so far (which in turn reflect the Eurasia-centrism of extant comparative folklore compilations), thus casts doubt on whether descent with modification along evolutionary lines is an appropriate model for how this folktale developed.

## Discussion

The analyses in the preceding section have shown three things.

First, variation between the different recorded versions of the Juan Oso story in the Andes do not pattern appreciably on geographical axes, other than that many (but not all) Southern Peruvian stories told in the region around Cuzco stand out by having features that do not recur elsewhere. Beyond that, the network shows little meaningfully interpretable spatial structure, in particular relatively little indications of “isolation by distance”-type spatial structure in which variation gradually increases with geographical distance. What evidence there is for such patterns is overlain by several cases in which stories recorded in the same place diverge so strongly from one another that the variation between them approaches the maximum observed in the entire dataset.

Second, variation in the story is not apparently linked to the type of Quechuan speech the stories are told in. Given the negative results of Redundancy Analyses, the stories appear to have developed independently of linguistic differences – this is consistent with the clear upper bound we can place on the time frame of the development of Andean version of the story in the early 16^th^ century, a point of time at which Quechuan already was a diversified language family.

Third and finally, different analyses converge to show that there is very little evidence for transmission of the story along classical evolutionary lines. Interestingly, though, the plot features that Join-Count statistics identifies as spatially clustered tend to co-occur in the same versions. If these are innovations (as the magic password, an element obviously transferred “laterally” from the oriental story of Ali Baba and the 40 thieves, suggests), then we could speak of shared innovations (synapomorphies) that are evidence for the development of shared traditions in a particular region. However, these cases are limited in number and geographical scope and seem to be an exception, rather than a general characteristic, of the overall way in which variation is or is not structured.

In sum, the usually investigated structuring principles of geography (an intuitively plausible proxy for the affordance of local and regional cultural interaction that may affect the way stories are told), linguistic affiliation (as a possible proxy for a broader cultural package oral traditions may be part of) and phylogenetic structure (as a manifestation of possibly shared histories of storytelling that are passed on from generation to generation) individually do not account for how the Juan Oso story has been recorded where it has been recorded in the Central Andes.

While for the underrepresented New World the comparative dataset is unusually large, the number of stories is relatively small when compared with the rich records of European and Eurasian folktales. Part of the explanation for the absence of significant relationships with geography and language may be the comparatively small number of tales and the sparsity of the dataset, which translate to low statistical power (even though recoding of features as multistate where this made sense did not lead to consistently better results). However, another part of the explanation may be qualitative and have to do with the specifics of Andean traditions of storytelling. I believe that the qualitative literature may help explain why we do not observe strong spatial structure; why, in contrast, stories that are very different from one another are told in the very same places; and why we might not see structure in the variation that is suggestive of development of innovations that are inherited and transmitted.

Like any good story, that of Juan Oso can be read on several levels. In its Andean context, Juan Oso is not just any story, but an allegorical comment on relations of Indigenous people to the institutions of colonial and modern society. These are represented by the malevolent priest, who is not just an impersonation of Catholic religion, but at the same time a wealthy landowner in a position of considerable worldly power (Weber, 1987). The theme of ethnic tension is also present in more subtle ways. As Allen (2002: 72) points out, the story plays with a characteristic ambiguity of the Quechua word *runa*. Roughly translatable as ‘human being’, *runa* and their society stand in contrast with the animal world, represented here by the predatory father bear. But in a narrow sense, *runa* also specifically refers to Indigenous people, and in this sense, *runa* and their mostly rural societies stand in contrast to the urban *mestizo* society that was formed in colonial times and that is represented by the Catholic priest. As Allen (2002) remarks further, in a subtle way, the story tongue-in-cheekly implies that *mestizos* and animals, both being non-*runa*, really belong to the same category of beings, with similar predatory and cunning characteristics.

Yet another possible reading relates to marriage relations and their intrinsic difficulties. The bear violently abducts the young woman, is sexually agressive, and an oppressive husband (indeed Allen, 2002 observes that Andean women, with reference to the story, may call their husbands, jokingly or not, *ukuku* ‘bear’). This same theme is brought to the fore in another story told to Allen (2002) in the southern Peruvian Andes, which tells of a condor who disguises himself as a refined gentlemen to propose to a rural girl. This story, as discussed by Allen (2002), plays with the fact that in-laws may move into their spouse’s household and eventually take it over when their in-laws pass – another case of cunning, predatory behavior of a non-*runa* animal disguised in human form. In the condor story, however, the girl learns of the true nature and intent of the condor and, like the abducted woman in the Juan Oso story, flees back home to her parents. There, they set up a trap for the condor in disguise, who ends up falling into and dying in a cauldron with boiling water.

We have encountered these plot elements already in several of the Southern Peruvian versions of Juan Oso, in which the father bear, rather than the condor, is trapped and killed in a cauldron in the house of the woman’s parent. It now turns out that these plot elements are actually a symptom of the Juan Oso story morphing mid-way into that of the predatory Condor. This is in fact also noted by Allen (2002), who refers to a version told to German archaeologist and ethnologist Max Uhle around the turn of the 20th century in Southern Peru.

As Allen (2002) notes, the condor story can be read as relating to man-woman and marriage relations in a way that is complementary to the Juan Oso story. In blending these two stories, the Andean storytellers who created the hybrid result show *agency* with regard to the traditional lore in interweaving the two narratives at a suitable point of articulation. This narratological entwinement has the effect of placing emphasis on a reading in which both stories relate to marriage and its dangers and tensions, rather than other possible ones.

One may surmise that further qualitatively similar entwinements are present in other parts of the story, with a similar purpose of highlighting aspects of the lore in the local, regional, or national social and political context of the storyteller and her audience. There is in fact evidence for this in the present dataset as well. The magic password that opens the cave in some versions of the Cuzco region is conspiciously similar to the story of Ali Baba and the Forty Thieves, where “Open Sesame!“ is the magic phrase that opens a cave in which thieves are hiding. In fact, in this case, one can see that the parallel brought to the fore by this move is that the father bear is in fact a thief of sorts, by virtue of his abduction of Juan Oso’s mother. Also variation in other individual bits of the story that we see as datapoints reflecting variation in the story – the background of the young woman, for instance– may reflect the ad hoc creative ways of storytellers manipulating creatively and alternating, perhaps on the fly, a general narratological frame. One potential case of this concerns a version of the story told in Anta in Southern Peru. In many Southern Peruvian versions, there is a colibri who informs the father bear that the woman has fled. However, in this version, it is the woman who a colibri informs that the father bear is pursuing her. Such inversions and transformations seem to be a characteristic of folktales more generally (Levi-Strauss, 1974), a characteristic that, narratologically, must be grounded in storyteller’s creative engagement, in their cultural context, with folkloric material.

## Conclusion

While I cannot fully engage with the rich folkloristic and ethnographic literature nor with the cultural evolution literature in its entirety, I believe that the observations made above may have implications for comparative approaches to folktales in the tradition of the “historical-comparative” school of folklore analysis, including those that have developed in the context of the cultural evolution movement. I present four points as a conclusion in the following, being aware that some may be so obvious and familiar to some readers that they might seem tantamount to carrying owls to Athens, whereas others may be felt to be questionable by the same or other readers.

The first concerns the question of representativeness of particular versions in light of intra-site and possibly even intra-speaker/storyteller variation in the arrangement and organization of motifs. To what extent a single specimen, fixed through transcription, can be considered representative of “the tale” as told in a particular locale, is a question of special import if recorded versions may be the product of ad hoc creative engagement of storytellers with motifs as they unfold their artistry on one particular occasion – a point made long ago by critics of the historical-geographical school of comparative folklore studies. According to Tehrani (2013), “folktales represent an excellent target for phylogenetic analysis because they are, almost by definition, products of descent with modification: Rather than being composed by a single author, a folktale typically evolves gradually over time, with new parts of the story added and others lost as it gets passed down from generation to generation.” The ethnographic perspective from the Central Andes suggests something different, namely that change is not always gradual but may be abrupt, and is specifically related to the narratological practice of individual storytellers. Modification may be highly context-dependent, and at the spontaneous discretion of a storyteller who adopts the lore to the current social micro-context of life in her community or the macro-social context of living conditions in 20^th^ century Peru. It is this agency in which we may find explanations for why some versions of the tale as told in the same place are as radically different from one another as ones from opposite ends of the country. At the same time, the Cuzco evidence suggests that modification in fact may be transmitted and amalgamate into a more fixed way of how the story is usually told – however, in this particular dataset, such patterns are very limited.

A second question of more general relevance is whether folktales can be considered adaptive, and in what ways. A classical position in cultural evolution is that functionally relevant items, e.g., parts of a canoe relevant for the functioning of the vessel (Rogers and Ehrlich 2008), evolve differently from ornamental designs as the former, but not the latter, are under selective pressure (Neiman 1995; Rogers and Ehrlich 2008). Consistent with this, Ross et al. (2013) assume that variation in folktales is “likely to have been predominantly selectively neutral (i.e., not ‘fuctional’ in the sense of being tested against the natural environment).” While it indeed is hard to imagine how folktale structure may be tested against the natural environment, it is much less implausible to suggest that it changes in response to their cultural environment by way of storytellers’ use of lore to make a point on current conditions or events. While cultural evolutionists are open to consider social contexts as a factor in the way folktales evolve (Tehrani 2023), to my knowledge the agency of narrators to employ and combine narrative material in ways that they function as commentaries on settings and events and in the family, local community, or society at large is not usually considered in this context.

A third point concerns methodological and conceptual assumptions. Advanced evolutionary models require the analyst to specify mechanisms for how the observed data is believed to have been generated. Typical parameters here concern the probabilities with which traits change their value; whether the same trait may be innovated independently along a tree, etc. The particular strength of Bayesian statistics (including but not limited to Bayesian phylogenetics) is that prior knowledge on the phenomenon under study can, and should, be incorporated into the formal model. However, as far as I am aware, the evolutionary models applied to folktale analysis do not take into account qualitative knowledge of how stories are told such as those I sketched above for the Central Andes.

More generally, comparative analysis of folktales in the cultural evolution framework are phylogenetic, whereas a more rhizome-like mode of development, in which stories may be rooted in several different antecedents, has been suggested already in the context of a debate from the 90s between representants of the nascent cultural evolution framework and anthropologists interested in the then much discussed notion of ethnogenesis (Moore 1994). The traditions of Andean storytelling reviewed above, like the cases discussed in Levi-Strauss (1974), suggest that rhizomatic models of folktale development may be equally or more appropriate than phylogenetic ones in at least these cases. At the same time, the case goes to show that the relevant phenomena cannot necessarily be modeled as “vertical transfer” or “diffusion” (by geographical distances as proxies or otherwise) as an additional component to “horizontal” transmission along evolutionary lines, but may require the specification of wholesale different models of development. To the extent that creative engagement with the material is considered licit behavior of storytellers (as opposed to ideologies that prioritize faithful transmission, e.g., in the case of the Homeric myths), these effects may be regularly observed, general characteristics of oral traditions and their development. (In addition, the decentralized semiotic connections between narrative plots which storytellers produce in this mode afford an intriguing link to modern literary theory: Deleuze and Guattari, 1987).

A fourth and final point concerns time depth. Based on associations with language phylogenies, it has been claimed that a select number of folktales can be reconstructed back to the Bronze Age (and, with or without formal analysis, mythological motifs have even been claimed to go back right to the dawn of humanity: Witzel, 2012; d’Huy, 2015). Among the analyzed datasets, the Juan Oso case study has the unusual property that an absolute time limit for onset of diversification can be set, viz. the first decades of the 16^th^ century (at least if one makes the simplifying assumption that a single version was introduced to the Andes from the Iberian Peninsula). As such, the study may have a role to play as a reference point for how much variation in oral traditions may be expected under specific conditions after a given amount of time, in this case ca. 500 years. It is well-known that biological evolution and cultural systems modelled as subject to evolutionary mechanisms, prominently language, develop at different speeds. This dataset may contribute to establish the relationship between variation in narrative traditions to their temporal development for those who wish to study them in a quantitative way, as cultural evolutionists do.

## Data Availability

All raw data and all R code used for statistical analysis are available on Zenodo at 10.5281/zenodo.16887588.
